# Supplementary material for: An exploratory study on midwives’ perceptions of the availability of preconception care in health facilities in the Kpandai District of Northern Ghana
Source: PLOS Glob Public Health. 2025 Aug 18;5(8):e0003850. doi: 10.1371/journal.pgph.0003850 (PMC12360517; doi:10.1371/journal.pgph.0003850)
Supplement: S1 Data — (DOCX) [file pgph.0003850.s001.docx]

**TRANSCRIBED DATA FOR THE STUDY: An exploratory study on midwives' perceptions of the availability of preconception care in health facilities in the Kpandai District of Northern Ghana.**

**DEMOGRAPHIC INFORMATION**

Interviewer: Can you share with me a brief information about yourself?

P1: Age: 42, Gender: Female, Marital status: Married, Religion: Christian, Education: Degree in Midwifery, Years of practice: 8 years, current grade: Senior Midwifery Officer

**KNOWLEDGE, ATTITUDES, AND PRACTICES OF MIDWIVES ON PCC**

Interviewer: What is your understanding of PCC?

P1: Is a care or education given to women before they conceive, like they become pregnant, so the education we give to them is the preconception care.

Interviewer: What are the components of PCC?

P1: So like when we are to educate them depending on the work the person is doing then maybe the age bracket too and whether the person is also married or not all these things also have to come in, because if maybe the person is a nurse, lets take it the person is a nurse then you have to check whether when you even deliver you will get a caretaker because you are not supposed to bring your child to the work side, whether you will get a caretaker or who will be with the child at the in case you are going for night duties, so all those things they have to consider before you try to get conceive or pregnant.

Interviewer: Still on the components, do you provide something like family planning services, screening your clients for sexually transmitted infections then the education, counselling giving supplements and those things, do you do them?

P1: All is very important yes, we do, because in case you have any condition that can prevent you from becoming pregnant you have to be sure of it. In case you even become pregnant and you are supposed to be on some medications, all those things are very import, yes

Interviewer: What are the benefits or outcomes of PCC?

P1: Yeah, it makes you well prepared before you get pregnant ahaa…. unlike the adolescents that they’re just doing their own enjoyment and by mistake they become pregnant and some will even try to abort but when you get this information it makes you well prepared….and ready to take care of your baby well.

Interviewer: Which category of individuals require PCC?

P1: I think errh every female who is up to the stage of reproductive age, everybody is entitled to especially like these supplements even at the adolescent age you can even start with the supplements that is why we go to the schools especially the JSS and SSS to give them the IFA then so as it prepares them towards conception.

Interviewer: Probe: Prioritizing people

P1: I think because of privacy sake they are not supposed to be added to the other group because you may likely especially the HIV if it is the diabetes they don’t have any problem but the HIV because when the baby is born the baby is supposed to be on medication so if they are sitting together and you mention it others will be alarm ahaaa..so it will be good if you get somebody like that you don’t really combine them with others to give the education.

Interviewer: What are the standardized procedures on PCC from WHO, MOH, GHS, and CHAG that you know of?

P1: I know there is a guideline but because we don’t normally have those classes, we just do it randomly in case somebody comes you that needs those services you educate as eerrh…maybe depending on the needs of the person, if not there are other guidelines that we can use that to educate, we don’t do that.

Interviewer: How do you receive clients seeking for PCC services in your facility? Probe:

P1: Yh, I think they are even the people we welcome more because it is scare to get such people coming closer, I remember a lady just got wedded within 1 month they came to me asking how they will do at least to also become pregnant yes so I was very happy just like you said earlier on…eerrh after educating them, I referred her again to see the doctor then doctor made her to do other labs and within some few months later she was pregnant so when she became pregnant, she herself she was always very happy coming for ANC, anything that happen to her even in the house she feel happy to contact me even at night because she saw the rapport build before the pregnancy so she knows that I also have interest in that pregnancy yes.

Interviewer: Where do you receive and provide PCC services to your clients?

PP1: No, as I said earlier on errh they come as they wish so because of that we don’t have any place for that. It was adolescent corner we created but its also not functioning well.

Interviewer: There is no time to provide PCC, what is your take on that as a midwife?

P1: There is enough time, it’s just that we don’t send the information so because of that they don’t also know that there are people to help them with that information.

Interviewer: PCC care depends on healthcare providers’ willingness, what is your take on that?

P1: Yeah…yes it is a responsibility, because when they come especially the family planning clients when they come we also add those ones to it , we will tell you maybe this method you are choosing in case you want to deliver there is no need that you come to us that we should reverse it for you, but in case you still…there are other methods you have to come back to us for us to for us to remove it before you become pregnant so because of that when they come we educate them on all those things.

Interviewer: Have you ever provided PCC to your clients? Explain your response

P1: Yes, I think I went on well, she was happy, she even came with the husband, aha…they just got wedded and came to me for consultation and they were very happy with that.

Interviewer: Have you received a client request for PCC counselling and consultation in the past 3 months? Or did you provide PCC in the past 3 months?

P1: In the past 3 months yes, somebody came, mmm somebody came, one teacher…., I referred her again to see doctor for other labs investigations, yes after the labs, she came back and told me everything was fine, yes.

Interviewer: What PCC services did you provide?

P1: Yeah…I only…I encouraged her about errh to feel relaxed because pregnancy is not by force, you can force God to give you pregnancy. Then I also educated here on folic acid intake, so I told her she should start errh taking the folic acid and I also referred her to doctor for some labs HIV, Hep, and errh the blood group, yes so she also did those labs and as for the diet and sex is that one and errh the menstrual period she should also try to monitor the menstrual period whether eerr how long the days and those things ahaa.. so that they should be able to capture when she conceives.

Interviewer: What role is there for PCC services in your practice?

P1: I think when you come to RCH everybody working there is entitled to, because people come, because those who are pregnant come, those who are also those who also want to prevent the pregnancy also come, so because of that everybody in RCH we provide the preconception care, but other staff I know with the obs we all learn in the school in our own way because as we said they come at will when many be you are staying with a relative and those things I think they contact them which is not official but I think in one way or the other the moment you are a health worker a lot of people may consult you pertaining to that. So, I think that is our role as health workers to also do that.

**DEMOGRAPHIC INFORMATION**

Interviewer: Can you share with me a brief information about yourself?

P2: Age: 28 years, gender: female, marital status: married with 2 kids, religion: Christian, education: diploma in midwifery, years of practice: working for about 5 years, current grade: Senior Staff Midwife.

**KNOWLEDGE, ATTITUDES, AND PRACTICES OF MIDWIVES ON PCC**

Interviewer: What is your understanding of PCC?

P2: What I understand about preconception care is a care is that, preconception care is a care or counselling given to couple that are preparing to become pregnant. So basically, you give them guidelines on how to care for their pregnancy till delivery.

Interviewer: What are the components of PCC?

P2: Some of the components of preconception care is that like erm….Ok usually we screen them we screen the couple to make sure that they are compactible as in maybe group blood grouping, then we also screen them like STIs, then we errn we counsel them on the need to maybe the need to prepare for the child maybe for the woman the need to prepare for breastfeeding that when she deliver she will have to breast feed you have to prepare her mind and all that and then maybe, medications that she will be taking during the pregnancy.

Interviewer: Any others?

P2: Yes, family planning too, we counsel them on family planning that is maybe after delivery after delivery they will have to counsel them to accept family planning services so that they can space their children, so that it will help them to take care of their children well.

Interviewer: What are the benefits or outcomes of PCC?

P2: Okay, usually they benefit a lot because, some of the clients are there who do not go through preconception care finally they when they finally realize that they are not compactible then it becomes a problem, because if we early detection of any sickness or maybe disease we will be able to counsel them so that we will take care of it before maybe they marry, so basically I believe they really benefit a lot during the preconception care.

Interviewer: Which category of individuals require PCC?

P2: Okay…preconception care usually couples who are not who haven’t given like haven’t given birth before yes, they are required to go through preconception care before getting pregnant and even if you have given birth, you can still come for a preconception care.

Interviewer: Probe: Prioritizing people

P2: Yes…especially the adolescents especially the adolescents we have to give them preconception care to prepare their minds of what they are going to errh become, because some of them are there if you don’t prepare their mind that if you deliver you will have to breastfeed your child, the person will not know or the person might not accept it, the person will not be ready to breastfeed the child if the person give birth. And then people like this errm those with the HIV too we have to also it is important they also we also do errm preconception care for them because already their immune system is down so you have to counsel them. Take them through preconception care before they become pregnant to make them aware that as they are becoming pregnant, it doesn’t mean that this their condition maybe if the person if you have the condition and you become pregnant and give birth your child will also become an HIV positive. We have to take them through and make them know that if you are if you deliver your child will be put on this, on this, on this or you yourself you need to be taking the medications so that the viral load will come down and that when you give birth your child will not. Some of them too are there if they don’t go through this preconception care, they don’t even have they don’t know how to go by their pregnancy… like they don’t know how to handle it, so they have to go through preconception care for us to tell them that they have to always attend errm ANC services regularly.

Interviewer: What are the standardized procedures on PCC from WHO, MOH, GHS, and CHAG that you know of?

P2: As for now I’ve not…there is no any protocol or guideline on that.

Interviewer: How do you receive clients seeking for PCC services in your facility? Probe:

P2: Usually, preconception care if somebody come to us for preconception care usually, we are always happy to receive the person because is not everybody that has that time to come for preconception care before becoming pregnant so anytime we receive somebody coming for preconception services we are always happy and we provide every information necessary to help the person.

Interviewer: There is no time to provide PCC, what is your take on that as a midwife?

P2: As a midwife, I think that is the problem of the day because a lot of people do not get time to do this preconception care to deliver this services but it’s necessary and important for us to because we need to get time we need to sacrifice if we really need want to do if we really want to do this preconception care services, we will be able to because I don’t think there is no time….is the problem

Interviewer: PCC care depends on healthcare providers’ willingness, what is your take on that?

P2: It should be a must because is part of the midwife role at the ANC, is part of the midwife role to provide preconception care to her clients, yes.

Interviewer: Have you ever provided PCC to your clients? Explain your response

P2: I have provided preconception care for a number of people at my facility here, just that it is not a regular thing, just that it is not a regular thing but I have provided for some, some come willingly to seek for information, assistance before they become pregnant.

Interviewer: Have you received a client request for PCC counselling and consultation in the past 3 months? Or did you provide PCC in the past 3 months?

P2: In the past 3 months, No…

Interviewer: What role is there for PCC services in your practice?

P2: So, as we earlier on said, I believe is a must, is must for the healthcare provider to provide preconception care especially the midwife yes, midwives are responsible for that for providing preconception care and is a must to provide those services.

**DEMOGRAPHIC INFORMATION**

Interviewer: Can you share with me a brief information about yourself?

P3: Age: 34 years, gender: female, marital status: single, religion: Muslim, education: Degree in midwifery, current grade: Midwifery Officer, years of practice: 6 months

**KNOWLEDGE, ATTITUDES, AND PRACTICES OF MIDWIVES ON PCC**

Interviewer: What is your understanding of PCC?

P3: Preconception is a care before pregnancy, a care given to woman before she conceive and in preconception too you need the two partners too to counsel them through and the counselling normally takes 1-3years before they will accept, the two will accept and it the counselling you need to know both particularly the woman psychological state, physical state, economic state and I think errm…physiological state, it all helps before the woman can conceive. Also, the counselling takes 1-3years before the two couple will accept. And before the woman will conceive too, she has to be on iron drugs or folic acid for the period of 3 months before she can conceive. Then if she accepts to be on the folic acid for 1-3 months and she conceive successfully then comprehensive or focus antenatal come in. Focus antenatal is in a lay man, 1 midwife 1 client. You begin by knowing the health status of the woman in the process you identify problems and then to help solve the problem till she is put on bed or till she delivers.

Interviewer: What are the components of PCC?

P3: So earlier on that is why I said that in preconception during the counselling errm, you need errm to take the two couple through errm, mentally yes so that you know, psychologically, physiologically, economically, so psychologically too we can talk about making the woman what… stress free and then the components could be during the counselling you have to know if there is any medical condition like diabetes, hypertension, sickle cell, HIV then you know what to do, I think this are the components then you even have to know whether they are compactible before ahaa, so I think those are some of the components.

Interviewer: Which category of individuals require PCC?

P3: Couples, thus male and females yeah

Interviewer: Probe: Prioritizing people

P3: Yes, yes the age groups of like 1- adolescents, 2- middle age like lets just say from errm or just 20-25 going……there are times these adolescents accept they will what they are planning of what bearing children so for them there is a need to given them attention and then good counselling for them to know because the maturity level is not all that high and the age group of 20-24 or going onwards.

Interviewer: What are the standardized procedures on PCC from WHO, MOH, GHS, and CHAG that you know of?

P3: I don’t have but normally within in the hospital protocol you need the midwife, a counsellor, and a physician.

Interviewer: How do you receive clients seeking for PCC services in your facility? Probe:

P3: We have to always smile, always be happy when talking to them we look in their faces and then we speak in what in a calmly manner and then I think we bring words that they will understand and then you should also be someone who is willing to help, not to always shout or during the counselling you will be shouting on them, you are harsh, no... because your attitude play a role, it helps the woman to open to tell you a lot.

Interviewer: Where do you receive and provide PCC services to your clients?

P3: Is a necessity, because it is the beginning of everything, so to get a full fetus, a healthy fetus, I think preconception errm there should be what a good preconception care to get a good and healthy fetus. So, in a healthcare setting there should be a designated area for preconception care services yes.

Interviewer: There is no time to provide PCC, what is your take on that as a midwife?

P3: And so that is why you earlier on asked whether it is a necessity or luxury so and I said Is a necessity because it helps to get a what a healthy baby at the end of what at the end of the what 280days of what pregnancy, do you understand, so I think they should be like midwives, doctors and counsellors who who are just at the preconception care unit to be doing that because when you look at errm client ratio you could see that 1 client or 1 midwife 5 clients, so the workload is the reason why we don’t give the care. But is a necessity because the beginning the conception will help errm identify abnormalities to intervene so that at the end the woman will get a healthy and normal what newborn or baby.

Interviewer: PCC care depends on healthcare providers’ willingness, what is your take on that?

P3: It is part of our responsibilities. That is when maybe during education, you educate the women to understand that well you don’t know that is why you conceive and you come for antenatal but if this is your first pregnancy, your next pregnancy there is a need to come for what counselling before you conceive and is the education. You go radios then you errh, you just educate the youth about that, so you see couples will what…willingly come and then see the midwife one on one oh! through your education we learned a lot and we are here for you to counsel us so the is the education that will help, radios, televisions, and those things so when they see it, they will help.

Interviewer: Have you ever provided PCC to your clients? Explain your response

P3: When I was in errm when I was in UDS my second year practically I was asked to educate a woman and I did that. I educated her on preconceptional care, what is, its importance.

P3: Interaction…yeah with the client, when I started is like she didn’t have any idea on what I was saying because she was a multip but per my education she to understand and through that she even told me that she lost she lost one of her pregnancy and the reason was is like a doctor said erm she and the husband were not compactible…so she had to leave that marriage and go for another and that one she errm they went to the hospital and they check and they said they were compactible, so I told her that is because of this care is not given.

Interviewer: Have you received a client request for PCC counselling and consultation in the past 3 months? Or did you provide PCC in the past 3 months?

P3: No...

Interviewer: What role is there for PCC services in your practice?

P3: It is our responsibility to provide a preconception care to help even reduce the workload. Because when you provide that preconceptional care, you can intervene at any point but where there is not that preconceptional care the woman conceive and comes and then there is like a lot of complications. There is a lot of complications and then it doesn’t reduce the workload, other than is either you sometimes try to what resuscitate the baby or you lose! A baby.

**DEMOGRAPHIC INFORMATION**

Interviewer: Can you share with me a brief information about yourself?

P4: Age: 29 years, gender: female, marital status: married, religion: Muslim, education: Diploma in midwifery, years of practice: 7 years, current grade: Senior Staff Midwife

**KNOWLEDGE, ATTITUDES, AND PRACTICES OF MIDWIVES ON PCC**

Interviewer: What is your understanding of PCC?

P4: For preconception care, I will say this are the care given to couples…those who have the intension of getting a child, the care you give to them, that’s what I will say, the care you give to them both couples which those that are preparing for a baby or to have a child that’s what I will say.

Interviewer: What are the components of PCC?

P4: You know when they come definitely you have to screen, you take their history’s as well. The history you can talk of social, occupation, then it maybe if the woman is having any conditions, not the woman per say both couple, both conditions sometimes I will give an example like this rhesus incompatibly issues, so the labs maybe the errh lets say the woman is O negative then the husband is O positive you know there is something we call rhesus incompatibility, so when you do this you will be able to rule those things out. And then the history that maybe any surgical history, any medical condition we know how to manage the errr the woman, maybe if she is a sickle cell patient at least your eyes have to be on her monitoring her all the time.

Interviewer: Any others?

P4: Family planning definitely yes, we have, because is the intension if you don’t make your mind to get pregnant definitely you have to protect you get something to protect yourself from you getting pregnant so definitely it is important.

Interviewer: Which category of individuals require PCC?

P4: You know the err the category that you are even talking, for the preconception care koraa even here koraa do we practice it?....is hardly you see or maybe those lets say though the women that are not they are not fertile seriously searching for pregnancy ahaa so maybe you will take the pain, you involve yourself in her shoe then you guide her or maybe some of them they can even approach you because they know you are a midwife oh this is the issue am having, I have married for so… long but am not getting a child so how will you help me, so that one maybe you get the opportunity to provide the preconception care services. But the categories maybe I may say that teenagers are also part, so these are the few this thing I know. Ahaaa, those with comorbidities like HIV, diabetes, hypertension oh yes you can also you can also do preconception care for them.

Interviewer: What are the benefits or outcomes of PCC?

P4: So the benefits, I will say it will help the mother for safe delivery and then aside that the baby too also comes ahaa at least when you do the preconception care monitoring them you would be taking good care of them at the end you may get a good results at least the woman may deliver an alive baby unlike those that they don’t care they don’t even come for antenatal, they don’t take these our routine drugs and other things and you know our routine drugs it helps. It can prevent a lot a whole lot of abnormalities this spinal bifida, hydrocephalus a whole lot ahaa, so when you do those things, it can help to prevent these abnormalities and then also help the safe delivery of the mother and then I may say it can prevent preterm labour are you with me or premature labor.

Interviewer: What are the standardized procedures on PCC from WHO, MOH, GHS, and CHAG that you know of?

P4: For the standardized you know the hospital because we don’t practice it it will be had ahaa it will be difficult. But maybe if you also know you teach me, we are all learning….

Interviewer: How do you receive clients seeking for PCC services in your facility? Probe:

P4: It depends, it depends upon the way the person will approach you, it will depend upon the way the person will approach you, some of them definitely you will feel for them, most cases that me I have ever seen with this preconception care is those that are in need of children aaahaa, so like when the person narrates her history or her this thing to you definitely you will feel for her ahaa so at least that kind of is it is it empathy or sympathy that I will I will even put? Ahaa so you have to put yourself in the person shoe then you help her out.

Interviewer: Where do you receive and provide PCC services to your clients?

P4: Oh yes…there should be a dedicated place and then maybe well-trained staff then you have to take them through what they are supposed to do.

Interviewer: There is no time to provide PCC, what is your take on that as a midwife?

P4: To me, I will only speak to where I am working, for here in fact we are lacking ahaa, we are lacking, because we are short of midwives you doing preconception care, it will be difficult, although you’ll do but it won’t be standard ahaa, unlike other hospitals that they have plenty midwives they get time for them and even anytime they call some of them can rush, Navorong where I did my service it was like that. The woman…. the midwife will just pick motor rush to the woman’s house and then any problem that she is having but just that she won’t deliver her in the house. Is the shortage of midwives, because I will come alone on duty deliver 6 women, attend to CS, NICU here but this thing medical ward the same, so you can imagine you are managing 4 wards in the same ward, one staff midwife on duty with national and you think you can have time for somebody coming for preconception care services.

Interviewer: PCC care depends on healthcare providers’ willingness, what is your take on that?

P4: Ahaa…is maybe if you if you want or how will I even say if you have the time then you have to, just like today like this only the ward is less busy like when you come and then am busy, I won’t get time.

Interviewer: Have you ever provided PCC to your clients? Explain your response

P4: One here and then one in Bimbila but may be it won’t be standard, just like I told you those that I’ve met most of them are in need of children so normally what I did was that I refer the person to doctor, you will refer them they will do screening and other things maybe some of them you’ll let her get some of the drugs that maybe some of them their hormones are too high ahaa….so this errr estrogen and progesterone so you know some of the hormones too affect child bearing so if it is low too definitely they have to get something, if it is high then you’ve to work on it. So, these are what I can remember.

Interviewer: Have you received a client request for PCC counselling and consultation in the past 3 months? Or did you provide PCC in the past 3 months?

P4: No…. fine me I will say maybe because there is no time for us, we the midwives we don’t get enough time to attend to them or how will I errh that’s how I will put we don’t get enough time, so you’ve come to work you are tired already the workload on you, so that is what I think.

Interviewer: What role is there for PCC services in your practice?

P4: Maybe other health providers can also come because you can’t do the work alone so preconception care shouldn’t be for only midwives at least other health workers too can also come in to help.

**DEMOGRAPHIC INFORMATION**

Interviewer: Can you share with me a brief information about yourself?

P5: Age: 33 years, gender: female, marital status: married, religion: Christian, education: diploma in midwifery, years of practice: 4 years, current grade: Senior Staff Midwife

**KNOWLEDGE, ATTITUDES, AND PRACTICES OF MIDWIVES ON PCC**

Interviewer: What is your understanding of PCC?

P5: Preconception care is like any care given to a couple or maybe partners who are in need of who wants to start giving birth so any care you give to them before they conceive that is preconception care.

Interviewer: What are the components of PCC?

P5: Some of the thing include some investigations to determine whether they are compactible, then family planning services and others like genetic counselling, screening for medical conditions, obstetric conditions.

Interviewer: Which category of individuals require PCC?

P5: The individuals or category of people who come for the services are couples, others too who are not married but they want to have children, people like adolescents.

Interviewer: Probe: Prioritizing people

P5: Yes, especially the adolescents, you know they are still schooling and they need to finish their education before they start maybe conception or giving birth so with those people you emphasize on maybe when they come for the family planning for them not to miss the date you give them so that it wouldn’t result in any unwanted pregnancy which might affect their education…… people with comorbidities like those with HIV, diabetes, hypertension…yes those kind of people we also have priority for them.

Interviewer: What are the benefits or outcomes of PCC?

P5: Some of the benefit includes certain things that will be prevented like if they hadn’t come for the services, like if they don’t assuming they don’t come for the services and they don’t know whether they are compactible or not and they happen to encounter challenges in the future that are some of the this things, and the adolescents too like I said it will also enable them to finish their school before they will start giving birth.

Interviewer: What are the standardized procedures on PCC from WHO, MOH, GHS, and CHAG that you know of?

P5: Yes…so just like in the place of the adolescents you don’t schedule them to meet maybe during ANC services or the time that others are there we have time for special time for those people because they are afraid that they might people might see then and that kind of stigma or something.

Interviewer: How do you receive clients seeking for PCC services in your facility? Probe:

P5: So, you first of all welcome them or you even great if they don’t great, you welcome them then you great if they don’t great then you give them a seat then you now enquire from them what they what to do from there you take it up from there if it is preconception care then you can provide it…….

Probe: are you always happy seeing such people?

P5: yes, am always happy considering the benefits am always happy.

Interviewer: Where do you receive and provide PCC services to your clients?

P5: It will be a necessity and that will also mean that we will have to educate the public to patronize it because just like I said earlier on some out of ignorance they don’t even know that there are supposed to come for preconception care so on that in that on that on the basis of that is a necessity.

Interviewer: There is no time to provide PCC, what is your take on that as a midwife?

P5: I will say there is you don’t determine when they come so there is no time anytime they come then you deliver the service.

Interviewer: PCC care depends on healthcare providers’ willingness, what is your take on that?

P5: I will say it is a must because is part of what we do, so somebody cannot come for you to give the care then you say you will not, you have to so I will say it is a must.

Probe: is it Part of your job description as midwives?

P5: Yes, is part of our job description as midwives.

Interviewer: Have you ever provided PCC to your clients? Explain your response

P5: Yes, I have ever provided preconception care, it was good because the person didn’t even know that it was a preconception care but per the interaction it was good and the outcome too was good.

Interviewer: Have you received a client request for PCC counselling and consultation in the past 3 months? Or did you provide PCC in the past 3 months?

P5: In the last 3 months yes.

Probe: How many time….

P5: I will say 3…

Probe: How was your interaction from the last one?

P5: the last one was someone who came, a nursing mother the child was 5 months old and she came she doesn’t want to become pregnant that early that the child is not grown, so I counseled her on what some of the methods that she can use as that time and she opted for a 1 month method which is an injection, that was what she took and went home and I rescheduled here.

Interviewer: What role is there for PCC services in your practice?

P5: I will say other health providers should also render the services because we come across those people everyday people who are in need of the services provided you can provide the service.

**DEMOGRAPHIC INFORMATION**

Interviewer: Can you share with me a brief information about yourself?

P6: Age: 26 years, gender: female, marital status: single, religion: Muslim, education: diploma in midwifery, years of practice: 2 years, current grade: Staff Midwife

**KNOWLEDGE, ATTITUDES, AND PRACTICES OF MIDWIVES ON PCC**

Interviewer: What is your understanding of PCC?

P6: So what I will say is that, with preconception care usually is a care that is rendered to women who intend to conceive not for now but probably the person has an intention of conceiving a year or two or more or when the person is married, and usually is not only meant for the women they can bring in their partners so we talk to them about what they have to do what they don’t have to do to help them carry their pregnancy without any risks or complications in the future, so that’s the little think I want to say about what preconception care is.

Interviewer: What are the components of PCC?

P6: We counsel them, then we run lab tests, then if they need further counselling from specialized people, you like refer them to those people as well, they are all part of the preconception care.

Interviewer: Which category of individuals require PCC?

P6: Usually for females who are within their reproductive age are mostly considered maybe if the persons is 18 we know that an adult is considered 18 and above so specifically you can say these are the category of people but as I said neither less men can equally be part because they are equally part of the process.

Interviewer: Probe: Prioritizing people

P6: Yes, we do because they will be regarded as high-risk clients who need special attention because when the person conceives it will not be only, she alone there is an additional being that is growing within her so there will be a need to counsel the person what to do and what not to do so that the baby that they intend bringing up would be safe.

Interviewer: What are the benefits or outcomes of PCC?

P6: So, with the benefits it ranges from a lot, let’s say carrying a pregnancy free risk to you being ready not only physically, but mentally ready, then you also get the free chance of asking all the questions who have so they are all benefits you can get out of coming in for that particular service.

Interviewer: What are the standardized procedures on PCC from WHO, MOH, GHS, and CHAG that you know of?

P6: Yes, we have…. with preconception care some might have the notion that oh they are afraid or something to confront you but everybody is eligible to have that care, so those guidelines are there what you have to do and what you don’t have to do, just to guide you give that service to your utmost best.

Interviewer: How do you receive clients seeking for PCC services in your facility? Probe:

P6: We are usually open and friendly to them because they always say your first impression counts so when the person comes in you welcome the person and make sure the environment is friendly or when a client come in and your face is maybe you frowned it, what the person intended to tell you koraa will scare the person so we always create a friendly environment.

Interviewer: Where do you receive and provide PCC services to your clients?

P6: Specifically, we don’t, we don’t have a unit that probably we’ve labeled it that for preconception care, so we add that service to our antenatal care so anyone that needs that service comes to us at antenatal for it. ….It will be a necessity if only they will make way for it because is good to get a unit for that because if the person is coming in and there are people around that kind of confidence to come in to tell you what she or he is coming in for would drive the person away but at least if you have a place that is for this when the person is coming the person knows that he or she is safe.

Interviewer: There is no time to provide PCC, what is your take on that as a midwife?

P6: Hmm…I think anytime can be dedicated or the right time for someone to come so you can’t say oh this particular day should be a time for preconception care day in day out people might just walk in to also seek for those services so I wouldn’t say we should have a specific time for anytime can be right for one to walk in for preconception care.

Interviewer: PCC care depends on healthcare providers’ willingness, what is your take on that?

P6: Is our right, responsibility to provide regardless your mood or whether even if you don’t want you have to provide because that’s your job description, so you can’t say maybe am moody am this it shouldn’t affect what you have to do.

Interviewer: Have you ever provided PCC to your clients? Explain your response

P6: Yes…

Interviewer: Have you received a client request for PCC counselling and consultation in the past 3 months? Or did you provide PCC in the past 3 months?

P6: I will say 4 hardly, they hardly don’t come but just once in a while.

Probe: How did you provide the care in one of them?

P6: We welcomed the person then usually you have to listen to the person client first what the client came in for before you can add up is a counselling session so you will have to listen more then give in your opinions and suggestions you will not dictate to the person you only guide the person on what to do then the person will pick the best option from it so that was how we went about it and it was successful.

Probe: What were some of the services?

P6: So, we counselled her about rhesus incompatibility, then we made her run lab tests went for scan and stuff so that was what we did for her.

Probe: The lab tests can you mention some of them?

P6: They went in for blood grouping, rhesus checking, syphilis, those STDs, HB too the person did that.

**DEMOGRAPHIC INFORMATION**

Interviewer: Can you share with me a brief information about yourself?

P7: Age: 34 years, gender: F, marital status: married, religion: Christian, education: Diploma in midwifery, years of practice: 4 years, current grade: Staff Midwife

**KNOWLEDGE, ATTITUDES, AND PRACTICES OF MIDWIVES ON PCC**

Interviewer: What is your understanding of PCC?

P7: I know preconception care is all the education, health benefits whatever you do before getting pregnant, whatever a client need to be done before the client gets pregnant. It comes together with the education you give, the nutrition, the medication the person would have to take the life changes everything before the person gets pregnant.

Interviewer: What are the components of PCC?

P7: Okay just as I said, life changes is one, nutrition comes in, medication also comes in, the persons errm how do I say it, exercise is also an aspect, basically yeah, yeah… and even counselling is also part, counselling also comes into it…

Probe: how about screening?

P7: screening yes screening is also part, screening yeah.

Interviewer: Which category of individuals require PCC?

P7: I think from the reproductive age everybody requires it. From the reproductive age let me just say errm I can’t I can’t limit to only teenagers……..With preconception care it is errm I would say anybody who wants to get pregnant, that one I would be opening the brackets too because if somebody is a teenager the person is not ready to be pregnant right now so I will actually close myself by saying erm if I should be giving the ages groups 20 going up *do you understand*, I stand to be corrected though.

Interviewer: Probe: Prioritizing people

P7: yes, yes, yes, because especially with what you’ve just mentioned HIV especially when the person gives birth a lot goes into it and the person would even need counselling before the person even gets pregnant because of the stigma and everything, the counselling would come in and then even during the pregnancy the medication the person would have to take and then right after birth the medications the child will also be taking, *do you understand*?

Interviewer: What are the benefits or outcomes of PCC?

P7: Okay, you see preconception care is very important is as important as ANC but is just that here in our domain we don’t really pay much attention to preconception care everybody is very very important, if if we were to be taking preconception care more serious here in Ghana I think a lot of infertility problems would have be solved or prevented because the person would start early and there are so many STI’s we don’t know we don’t even know about it before the person would know it it would have already caused havoc to the person’s uterus so one of the important, one of the benefits of the preconception care is curbing infertility, reducing infertility if that is how I should put it. We also use the opportunity to give education if the person start preconception care early it will reduce the work of the antenatal because we will be doing more of the education in the perception session or period, so more of the education the person is already aware so you wouldn’t spend much time on the education there, and even when it happens which will fall into folic acid when you want to take it before even getting pregnant, you have to take it like 3 months before you even get pregnant so that your body gets storage of the folic acid, so when the person gets pregnant the person has more stores of the folic acid because they said when the person gets to during pregnancy at 3 months the person is supposed you are supposed to stop taking the folic acid because it has already finished its work on the fetus, yes. So, when we do preconception care it can also reduce some congenital malformations such as the neural tube defects, which is caused by folic acid deficiency, so if the person starts taking the folic acid before getting pregnant some of these congenital malformations would be it wouldn’t even be found yeah.

Interviewer: What are the standardized procedures on PCC from WHO, MOH, GHS, and CHAG that you know of?

P7: Hmm…okay to be frank with you I haven’t gone to a facility which is providing preconception care before with the protocol, I wouldn’t really know how it is done. I think education is one, we have a place for education, we can have time for the screening which we spoke about yes, HIV is also Hep B is also part to know the persons status and the person can also be referred to a nutritionist…these are some of the things preconception cares would be needed. So, we can give medications, the screening comes in, education also comes in, now koraa they say we shouldn’t call it education but we should call it counselling, ahaa, so the counselling comes in yes yeah…. I haven’t really seen a standardized protocol before, this one am just giving my opinion for treatment for a patient.

Interviewer: How do you receive clients seeking for PCC services in your facility? Probe:

P7: If our government certain we have it would it would be how do I put it errr, it would be nice to have people coming for preconception care even though we don’t have it I have haven’t seen somebody and is just that is lack of the education that is why they don’t come because somebody doesn’t even know they have to do something before they get pregnant, they only come when let’s say they have tried for a couple of years and pregnancy is not coming then they come and check the reason. So I think it would it would be fine, okay let’s personalize it, if somebody comes to my facility and tells me she wants preconception care it will give you the opportunity and it will save us so it will save us so much time in as in 4, 5 years the person is not getting pregnancy, she has done a lot of things before, so I think its fine it would be nice if they walk into our facility and want preconception care because what we are already doing at the ANC and the other services we offer but hardly do you see clients coming in for preconception care.

Interviewer: There is no time to provide PCC, what is your take on that as a midwife?

P7: I think there is time is all about is all about strategizing, is just like we having a section for ANC and because we have a section for ANC there is a personnel there, so if we have a section for preconception care there is definitely going to be a personnel there right?, yes, and we can strategizes it just as the ANC services, the personnel may go Monday to Friday and rest during the weekend just as we have the ANC and the family planning services, I think so

Interviewer: PCC care depends on healthcare providers’ willingness, what is your take on that?

P7: Preconception care is a responsibility because we are been taught in school yes, I think is a responsibility not a luxury, yeah.

Interviewer: Have you ever provided PCC to your clients? Explain your response

P7: We actually, I think in an informal way we just the person just comes by way of discussion then you channel to preconception care so is like an informal way of rendering service and you end up prescribing folic acid to the person which is one of the components of preconception.

Interviewer: Have you received a client request for PCC counselling and consultation in the past 3 months? Or did you provide PCC in the past 3 months?

P7: The last 3 months yes, yes... I think about two clients,

Probe: Tell me how you went about it.

P7: okay so, I think just as I said earlier is just about getting married and not conceiving for some time so it becomes a boarder and a worry so she came to me and then spoke about it that and we even as we were speaking I got to know that her menses wasn’t regular, so I had to prescribe folic acid and iron tablet for her so that the following, I told her to finish taking the drug then she come back but she didn’t come back and I also didn’t go and do a follow up. And then the second one, the second one also wanted to get pregnant and by way of discussions they actually indicated they wanted to get pregnant but its not coming for some time then you turn asking them questions lets say the number of times they meet their partners because this second client she has deliver 4 and wants another baby, so I had to delve into why she is not but she had taken, I even got to know she was doing family planning, so I had tom tell to stop and wait for sometime yes and she came to she is pregnant now today yes.

Interviewer: What role is there for PCC services in your practice?

P7: I think is not only the midwife role because a lot goes into it which we have the screening, the screening may not just be done by the midwife, the counselling the counselling even though we are trained but we have trained counsellors, so I don’t think is basically just the role of the midwife even though we are the first people the clients come to, but it has to be is a team work, so there should be a team for it, so the client meets this person, goes here for this, goes here for that, goes here for this, yes, a dietician is also part, a nutritionist is also part, a nutritionist or a dietitian for diet and all that, yes and sometimes the doctor even come in ahaa yes.

**DEMOGRAPHIC INFORMATION**

Interviewer: Can you share with me a brief information about yourself?

P8: Age: 37 years, gender: female, marital status: married, religion: Christian, education: degree in midwifery, years of practice: 6 years, current grade: Midwifery Officer

**KNOWLEDGE, ATTITUDES, AND PRACTICES OF MIDWIVES ON PCC**

Interviewer: What is your understanding of PCC?

P8: Ok is the care we give to ladies in their reproductive age before conceiving.

Interviewer: What are the components of PCC?

P8: So, basically, we educate them on hygiene, nutrition, then if possible if they are to take some drugs before conceiving… for the reproductive health in general so they can come for preconceptional care so that we educate them on maybe some reproductive hygiene.

Interviewer: Which category of individuals require PCC?

P8: I think every woman within their reproduction age can come for preconceptional care, like if I should give age from 18 years to 35 years or yeah

Interviewer: Probe: Prioritizing people

P8: No, no anybody at all who come.

Probe: how about infertility, commodities….

P8: Oh no yeah, for such people yes, we definitely have priority yeah because they need special attention and special care yeah.

Interviewer: What are the benefits or outcomes of PCC?

P8: Sure! one it helps in safe safety in throughout their pregnancy and delivery and also it hep them like let me say spacing the woman will know when to get pregnant and when not to, so is also I think is also a benefit yeah, then it will help them have healthy pregnancy.

Interviewer: What are the standardized procedures on PCC from WHO, MOH, GHS, and CHAG that you know of?

P8: I will say no, like we don’t have standardized protocol or guideline because we don’t normally practice it, but when somebody comes to maybe consultors for preconceptional care that one we give, so we give it the way we understand but not following any standardized guideline or protocol.

Interviewer: How do you receive clients seeking for PCC services in your facility? Probe:

P8: We welcome the person and whatever knowledge we have about preconceptional care we give to the person.

Interviewer: There is no time to provide PCC, what is your take on that as a midwife?

P8: I think especially for here our hospital or our unit I will say lack of staff, like you’ve seen today am the only person on duty, so imagining taking care of maternity, labor ward, and other stuffs plus even our neonates and people are lined up here for preconceptional care it will be difficult so I will say in our unit, I will say is the lack of staff.

Interviewer: PCC care depends on healthcare providers’ willingness, what is your take on that?

P8: I will say it should be a responsibility because at the end of the day when the woman conceive definitely the person will come and land in your hand you have to take care of the woman so if you give preconceptional care you are your talking about the persons hygiene everything inclusive so if the person is having a healthy pregnancy it won’t bring workload on midwives so I think is our responsibility.

Probe: Is it part of your job description?

P8: Yes, as a midwife is part of our responsibilities yeah.

Interviewer: Have you ever provided PCC to your clients? Explain your response

P8: Yes, I have ever provided preconcetional care services to many clients.

Interviewer: Have you received a client request for PCC counselling and consultation in the past 3 months? Or did you provide PCC in the past 3 months?

P8: No, is more than 3 months….

Interviewer: what about your previous interactions….

P8: ok, so I think the client came yeah and we talked about health, nutrition, then we talked about hygiene yes then I talked about especially the readiness yeah if the person is really ready to go through pregnancy, ready with finances and all that yeah so I gave that I think that aspect then I told the person if she is ready then we’ll talk about maybe if there is the need for medication especially folic acid that is what we always advise them to take before conceiving yeah……

Interviewer: What role is there for PCC services in your practice?

P8: I think every midwife yeah, every midwife is supposed to, I think every midwife is supposed to provide preconceptional care because even in school is part of our courses that we learn so is supposed to be midwives I think…….

Probe: other HCP

P8: Ok, so definitely when the person come there will be something like lab and if there is issue you include doctors so they can also play part in preconception care.

**DEMOGRAPHIC INFORMATION**

Interviewer: Can you share with me a brief information about yourself?

P9: Age: 28 years, gender: female, religion: Christian, marital status: single, education: diploma in midwifery, years of practice: 2 years, current grade: Staff Midwife

**KNOWLEDGE, ATTITUDES, AND PRACTICES OF MIDWIVES ON PCC**

Interviewer: What is your understanding of PCC?

P9: Preconception care is like normally we is a care that we give to couples or any other individuals like lets say even if you are not married and you are ready like to get pregnant, mostly is not only couples like individuals or partners who are ready to like get pregnant, we counsel them on certain stuffs so that they will prepare themselves and make the right decision towards pregnancy, so basically that is what I can say.

Interviewer: What are the components of PCC?

P9: Ok we do nutritional counselling, genetics, psychological counselling, then yeah yeah well for now that what I can say…. laughs…

Probe: What about family planning?

P9: Yeah, family planning too is also part of the component?

Probe: What about laboratory screening.

P9: Yes, we sometimes they go through this thing rhesus factor is part of the this thing that we do, and then the blood group is also part then sickling is also part of the lab tests err.. Hep B, HIV, G6PD, sometimes too Hb is also count.

Interviewer: Which category of individuals require PCC?

P9: Couples those that are married and we have those that are also like err…they are staying together but they are like they are not ready to get married but they want to give birth.

Interviewer: Probe: Prioritizing people

P9: Yes, we have, especially those who comes with conditions like some health conditions, like let’s say sickle cell, hypertension, HIV clients yeah, those people they are special clients and errm someone like let’s say nullipara like the person is advanced in age but has never conceived before ahaa they are also part….

Probe: About adolescents?

P9: Yes, errm the adolescents yeah, we do…laughs…

Interviewer: What are the benefits or outcomes of PCC?

P9: The benefits, it helps…laughs… they get because there are times they some will come around and you take them through the counselling then issues pop out like things they didn’t know about let’s say like the sickle cell for example some usually don’t go for this thing err screening so through that they are able to detect that oh am a carrier or am a sickle cell this thing so when they get to know that they are able to plan on how to take care of their unborn child incase the child comes out to be a sickle cell this thing ahaa so that is another benefit. For the benefits there are a lot of benefits, they are able to take of their children, they themselves they know like the stages like that comes like with pregnancy like the way we have first stage what first trimester what goes into it, second trimester, third trimester and those stuffs, for now that’s what I can say.

Interviewer: What are the standardized procedures on PCC from WHO, MOH, GHS, and CHAG that you know of?

P9: We don’t, there is no protocol……there are guidelines but protocols…. not readily available…yeah let’s skip it.

Interviewer: How do you receive clients seeking for PCC services in your facility? Probe:

P9: We relate to them like just any other individuals that comes around, there is no like attitude or any other this thing attached to it….

Probe: Are you happy?

P9: yeah, and you know this our sector is is not common like that like err other places, hardly like if they will come koraa most a times is the the err women that will come then during the conversation then you now invite the man to come, hardly will you see them coming together yeah….

Probe: How is the communication?

P9: friendly…. laughs…is like one-to-one interaction is not like you would just have to pump everything into to them no, is a counselling session and that one you don’t have to impose certain things on them, they will bring their opinion then you take it up.

Interviewer: Where do you receive and provide PCC services to your clients?

P9: I do it with the community health nurse, and for here, for my facility we don’t have lab so when we are done when we get to the labs side, we refer them.

Interviewer: There is no time to provide PCC, what is your take on that as a midwife?

P9: That’s another challenge, the staffing is also another challenge, currently where I’m am the only midwife, so let’s say in a day that it will crush with when the community health nurse is having CWC or home like going out for outreach and there is pressure on me, the, attending to those clients is always difficult because I’m alone.

Interviewer: PCC care depends on healthcare providers’ willingness, what is your take on that?

P9: Yeah, it is, is part of our job this thing so we are willing….no as and when the client comes, is not as when you, you want to provide no, it depends on the clients, so anytime they come you have to get time for them.

Interviewer: Have you ever provided PCC to your clients? Explain your response

P9: Yes I did and it came out successfully, errm there was this couple that came around when I was posted and the issue was that there anytime the lady picks seed she get miscarriage so they came around then I took them through the counselling I made them to go for lab investigation and stuffs, and when the results came it was due to rhesus factor ahaa, so I referred them and they too they were the understanding couple they went through everything and thanks be to God now they have a baby boy who is getting to 1 year now.

Interviewer: Have you received a client request for PCC counselling and consultation in the past 3 months? Or did you provide PCC in the past 3 months?

P9: No….

Probe: why?

P9: laughs…that one I don’t know, I can’t answer…. laughs…

Interviewer: What role is there for PCC services in your practice?

P9: As I earlier on said when you the components of preconception care, lets the nutrition aspect for instances, you need a dietitian, and then the lab aspect you need a lab technician, so is not for only midwives’ other health workers need to get involved….

Probe: How about other staff?

P9: I’ve a community there is a community health nurse, field technician and then general nurses….

Probe: Dose all of them provide PCC?

P9: For now, is just the midwife and then the community health nurse for now.

**DEMOGRAPHIC INFORMATION**

Interviewer: Can you share with me a brief information about yourself?

P10: Age: 34 years, gender: female, marital status: married, religion: Muslim, education: Diploma in midwifery, years of practice: 5 years, current grade: Senior Staff Midwife

**KNOWLEDGE, ATTITUDES, AND PRACTICES OF MIDWIVES ON PCC**

Interviewer: What is your understanding of PCC?

P10: Preconception care is a care given to a woman whose who want to get pregnant, so is a care that you give it to her to prepare her psychologically, emotionally, and then the body.

Interviewer: What are the components of PCC?

P10: It depend, we have the ages varies, maybe somebody at 20, 18 years or somebody too maybe after 40 or 35 years ahaa so most of them you take them through some labs then you give them some supplements vitamin supplement like folic acid and those things for the person to take, and most of them too mostly some are there they give with their husbands but this our northern section is mostly the ladies that come for the care…..

Probe: How about family planning?

P10: most of them it depends, if maybe the person is having a problem with the menses cycle ahaa so maybe the person is having irregular menses or maybe is not even coming most of the family planning too corrects those things example like norigynon when somebody is having a problem with the menstrual cycle norigynon normally most of them they use norigynon to correct it.

Interviewer: Which category of individuals require PCC?

P10: The err those like after 18 years and the person has just gotten married then want to get pregnant and you know you have to take the person through some education and then running labs mostly especially the health concern, and you know where to take if from, and then elderly primid after 35 years that is the time the person is struggling to get her first pregnancy those people is very necessary…

Interviewer: Probe: Prioritizing people

P10: Those people are the after 35 years struggling to get her first pregnancy or maybe the person too have gotten one and is taking maybe 7 years, 8 years the person has not gotten another one….

Probe: How about comorbidities?

P10: Yeah, those ones too are for them koraa even when they are even pregnant, even if the person come during the pregnancy and you detect it you’ve to pay attention to it. but if maybe the person is retro positive, Hep B positive and then diabetes during the preconception care you’ve to let the person know maybe if it is HIV you start the person starts the drugs before the conception ahaa, if it is the Hep B you let the person know what will happen after delivery or during pregnancy and things to avoid, and then the diabetes too the same thing, but mostly especially the HIV most of them koraa don’t even want their partner to know ahaa.

Interviewer: What are the benefits or outcomes of PCC?

P10: Yeah, it makes them to prepare psychologically, the body everything even financially, they are able to prepare because during those time you tell them everything, when you get pregnant what is possible to happen ahaa so they prepare everything down so during when they conceive then they don’t have most of the difficulty because they already know what is involved.

Interviewer: What are the standardized procedures on PCC from WHO, MOH, GHS, and CHAG that you know of?

P10: Mostly when we came here, they were saying they have adolescent units, mostly it was that place they used to take them, but now is like is not working, but those they want normally they just come and approach a midwife, oh this is what I want then the midwife too will take you through if she can’t then she also refers you to somebody else. Me I’ve not seen it if they have then I’ve not seen it, maybe because am not at ANC too.

Interviewer: How do you receive clients seeking for PCC services in your facility? Probe:

P10: Oh, I welcome them and then educate them more about it, then you let them know the preconception care is not just talking to the person like this, you let the person what to do ahaa, especially starting from the labs ahaa, per the labs you’ll know how to handle the person. Mostly koraa especially these hormonal imbalances all those things they do it so per the labs you’ll know how to handle the person….

Probe: Are you happy?

P10: Oh! We’re always happy to receive them especially when you’re able to take the person through and see the person happily pregnant and deliver you yourself your happy, and wherever the person too sees you that respect is there.

Interviewer: Where do you receive and provide PCC services to your clients?

P10: No, that one di33 I can’t point that this is the unit.

Interviewer: There is no time to provide PCC, what is your take on that as a midwife?

P10: No, that one di33 is a lair…then if you don’t have time to provide preconception care then you won’t get time too for ANC….

Probe: Is there enough time?

P10: You’ve to get time for the client, if you get time for them during preconception when they are pregnant you don’t even suffer.

Interviewer: PCC care depends on healthcare providers’ willingness, what is your take on that?

P10: No it depend, let’s say if it is me maybe I’m on duty I’ve come the place is busy oh madam you see the place is busy let’s schedule it to this time fine but because if you are busy like here am just alone as a staff here the rest are students so as am sitting your mind is somewhere you would even give the person the care or the education you want to give to the person, the person will not get it but its just between you and the client oh madam this time am busy let’s schedule it to this time so that we’ll get enough time for ourselves, so depend on how you people will agree ahaa…..

Probe: Are you supposed to deliver PCC as a midwife….

P10: Especially when the person come to approach you, you’ve to do it.

Interviewer: Have you ever provided PCC to your clients? Explain your response

P10: Yes…but finally I’ve to refer her to teaching hospital tamale, yeah it was about this her menses they said they want to conceive so we do all what so finally we did the hormonal tests and there was some problem so I referred them to see one gynecologist at Tamale so currently they are seeing the gynecologist at Tamale, last month they went….

Probe: The last interaction did it occur within the last 3 months?

P10: this is the second month; we started here 2 months but it wasn’t changing so they went to Tamale this is the second month it was February they went so the man gave them their next visit and that will be twenty something much….

Probe: what other services did you provide as part of the PCC?

P10: because initially they came with not getting her menses but anytime she do pregnancy test is not pregnancy, so we started with norigynon when she did the norigynon one month time the menses came, so I advised them to take the second dose to correct it but the guy was saying they will not take because is a family planning this thing they want to try to see so I kept them on folic acid for them to take and then there is one supplement too “prowoman” so when they took it the next month she had her menses again but when we run the labs the prolactin was high which is supposed to be high during breastfeeding, so that was why I referred them to Tamale Teaching Hospital to see a specialist there….

Probe: Did you do blood grouping, rhesus factor checking?

P10: They were all okay.

Interviewer: What role is there for PCC services in your practice?

P10: Yes, is a responsibility for a midwife to do preconception care before you now go to antenatal and then delivery and then puerperium so ideally, we supposed to start from preconception care so when the person now conceive you start from the antenatal care before during antenatal care you prepare the person for delivery after delivery for puerperium, so all those things are for midwives so is not for any other healthcare provider…

Probe: Is it only midwives that deliver PCC?

P10: yeah, even the antenatal koraa is only midwives.

**DEMOGRAPHIC INFORMATION**

Interviewer: Can you share with me a brief information about yourself?

P11: Age: 29 years, gender: female, marital status: Single, religion: Muslim, education: Diploma in midwifery, years of practice: 1 year, current grade: Staff Midwife

**KNOWLEDGE, ATTITUDES, AND PRACTICES OF MIDWIVES ON PCC**

Interviewer: What is your understanding of PCC?

P11: Ok, preconception care is just like you preparing your client or a pregnant woman, lets say a client psychologically, mentally, physically before pregnancy and it deals with you counselling the client making her know her status whether errm she is errm she knows her blood group, HIV status and then you a test to know whether it is positive or not and moreover if we’re to do you go in for a scan for everything to be successful before you now enter into pregnancy.

Interviewer: What are the components of PCC?

P11: As I said earlier you counsel, you prepare the woman physically, mentally and psychologically, because her mind has to be a stable, she has to get a stable mind before she enters into conception, and we run test on her, we counsel her, we make her know the process she is going to go through or the journey she is embarking on because is a long journey 9months is not any playful journey it’s a journey on its own and it’s a human you are going to carry in you, so you’ve to prepare your mind towards whatever will happen before the conception or during conception…

Probe: How about labs?

P11: As I said you have to know your blood group, then you know your status whether you are an HIV positive or negative and then you know your hepatitis B status as well, and then we do abdominal scan to verify whether your reproductive structures are normal or has any abnormalities, we also check to see your sickling status.

Interviewer: Which category of individuals require PCC?

P11: We don’t have any category and besides we normally deal with married people and even if you are not married, we prepare your mind towards it because a person is going to get married definitely, you’ll conceive ahaa so we’ve to prepare your mind, we’ve to prepare you towards it and we know the consequences or we have the advantages and we’ve the disadvantages.

Interviewer: Probe: Prioritizing people

P11: Yes, yes, yes is through we have errm as for the adolescents you know they don’t always know much though they’ve heard they don’t know what it entails so you’ve to sit them down you advise them, you give them the necessary erm support they need during preconception and conception, the knowledge about it, you have to impact some or let them know the importance of it and when it comes to the adults you know some are in their 30s and it might be their first child they are looking for so these people you don’t joke with them you take them through thoroughly because errm upon research when you age like when you are in your 30s and lets say 35 going errm beginning is always difficult is sometimes difficult for some of them because they said your fertile period is mostly in your 20s ahaa so if you are in 20s and then you’re able to conceive its always ok than you been in your late 30s and this they go errm when you are in your 30s too or your late 30s there are complications or lets say at times there are complications attached or occur during pregnancy so you’ve to take them through and let them know and people like that their babies they are most they’re always special to them so you also have to treat them special for them to know that yeah age doesn’t matter ahaa it doesn’t matter whether you are 30s you are old err you are in you late 30s or so, you give them the care that they need, you take them through well let them know what to do and what not to do.

Interviewer: What are the standardized procedures on PCC from WHO, MOH, GHS, and CHAG that you know of?

P11: Herr…let’s say when you come to health sector or our side errm we always encourage male involvement, so lets say if there are clients here and lets say the person is even coming for an ANC and she came alone and a different client who comes with the husband we’ll attend to the one who comes with the husband first before but not all that, the protocol doesn’t cover only that errm we also look at the health aspect lets say if you are pregnant you know pregnancy varies in individuals, you can be pregnant and be active and some too they don’t, like they don’t have that strength to condone or to keep it up to term, at times some they feel weak some they even have to be at the hospital throughout so we prioritize them and we lets say let me come to the your status lets say if the person is HIV or hep B or this you know some don’t want people to know that yeah they have it, so we treat them and you’ve to let them come on a special day for you to take care of them let them know what it all entails so I think that is it.

Interviewer: How do you receive clients seeking for PCC services in your facility? Probe:

P11: Is a good idea because if the person don’t believe in you there is no way the person would come and even this is always a privilege for you the person has given you the maximum respect an the person has trusted all her hopes in you so you don’t have to disappoint her you also have to give in your best to let her know yes she is most welcome anytime any day, you receive her warmly, you give her the best options and the best advice or counseling ever that she needs and you don’t oppose or impose, you assist her in doing the right thing.

Interviewer: Where do you receive and provide PCC services to your clients?

P11: Yes, even coming err, a person coming to you for you to test whether she is pregnant or not or if the person comes to tell you her problem it's all preconception let's say errm she has missed her menses like how should I even put it, yes, so if the person comes to you it’s all preconception, the person comes to you want to know you do test you make her go in for a scan, you give her the best counseling ever it's all preconception because you do not know, you haven’t done the test yet to confirm whether she is pregnant or not so you are preparing her towards the pregnancy so I think its preconception care…practical services…ok, you know errm you first assure the client let her know because she have to have trust in you and you let her know that what you guys are doing its between both like you, so the privacy and confidentiality have to come in, and you counsel her you ask her of her menstrual cycle, her last err month or LMP so you know whether she like the month she had her menses and the month it didn’t come ahaa and even that you’ve to know her background because psychologically background the she has to be stable and the, the background too also help and the kind of activity she is into ahaa her work errm her daily activities you have to know so that you know how to counsel her on whatever you guys are discussing on.

Probe: How about supplements?

P11: Yes, yes because we always give folic acid and multivite because every woman you have to be taking it because of the monthly cycle, it prepares us and it makes us fertile as well ahaa, so and not only that you have to check your HB to know your HB level whether we’ve to prescribe certain drugs for you to bee on or not so it’s all inclusive and as I said earlier we do test to know your HB, Hep B status, HIV status, so let’s say if you’re an HIV we now take you, we give you the counselling and then we take you to a higher facility for them to render the services for you.

**DEMOGRAPHIC INFORMATION**

Interviewer: Can you share with me a brief information about yourself?

P12: Age: 37 years, gender: female, marital status: Married, religion: Christian, education: Diploma in midwifery, years of practice: 4 years, current grade: Senior Staff Midwife

**KNOWLEDGE, ATTITUDES, AND PRACTICES OF MIDWIVES ON PCC**

Interviewer: What is your understanding of PCC?

P12: Preconception care is render to couples or adolescents or women in their reproductive age prior to pregnancy to prevent or manage any risk identified or that could arise during pregnancy.

Interviewer: What are the components of PCC?

P12: With the preconception care, we do genetic counselling to the couples or women in their fertile age, then we run some labs which entails a lot maybe the urinalysis, semen analysis, diabetes, whether the person is having any complication or hypertensive disorder already or which may arise during the pregnancy period, then you take their height too and their weight to access them, so that’s what I know that goes into it.

Interviewer: Which category of individuals require PCC?

P12: The requirement of people, it could be couples, it could be adolescent girls or adolescents, it could be women in their fertile age or reproductive age…

Interviewer: Probe: Prioritizing people

P12: For health delivery it should be equal but if some comes with a peculiar or a risk factor then more attention is given to such group than the others…..yes, much attention is paid to them because if you don’t deal with them with much attention to identify most of these problems you might end up losing the mother or the baby or at the end the client might not get the results they’re looking for….

Interviewer: What are the benefits or outcomes of PCC?

P12: Like let’s take those with HIV for instance, when you come for preconception care and it is identified or you are already aware that you are a positive couple or the woman is positive or the man is negative then the one who is positive you put the person on anti-retroviral therapy so that the viral load may or should come down before picking seed to prevent the transmission to the unborn baby, and maybe with the err a client with hypertensive conditions when you are able to identify them you know how to manage them to prevent further complications during the pregnancy period.

Interviewer: What are the standardized procedures on PCC from WHO, MOH, GHS, and CHAG that you know of?

P12: There is no protocol but from school knowledge that’s what we use to provide the preconception care.

Interviewer: How do you receive clients seeking for PCC services in your facility? Probe:

P12: Is just like any other healthcare delivery we’re giving out so we receive them with the way we’re supposed to receive clients……

Probe: Are you happy about it?

P12: We’re happy to receive them.

Interviewer: Do you have a dedicated place where you receive and provide PCC services to your clients?

P12: No, but at our antenatal services.

Interviewer: Have you ever provided PCC to your clients? Explain your response

P12: Yes, it was good this one, she had ever delivered before but the gap was very long, long interval so she was now seeking to conceive again so we were trying one on one, one on one and later on she conceived…..like for instance we asked her about her menstrual cycle, we took her history about her menstrual history then we calculated when she ovulate, how she have to meet with the husband ahaa, so although she was trying it, when she goes to the husband’s place and come then she will just return to her normal menses again so we asked her to stay back maybe she might be picking it unknowingly ahaa, so this time around she stayed back the husband came she didn’t travel and now she is having the pregnancy, then she was also kept on daily folic acid.

**DEMOGRAPHIC INFORMATION**

Interviewer: Can you share with me a brief information about yourself?

P13: Age: 32 years, gender: female, marital status: Single, religion: Christian, education: Diploma in midwifery, years of practice: 3 years, current grade: Senior Staff Midwife

**KNOWLEDGE, ATTITUDES, AND PRACTICES OF MIDWIVES ON PCC**

Interviewer: What is your understanding of PCC?

P13: When we talk of preconception pre means before pregnancy therefore when a person approaches a midwife as the first time a couple coming to you to discuss ways to get pregnant things to put in place to get pregnant, the necessary medications that they need, the necessary advice, things that will happen like she basically wants to know more about pregnancy. So, the preconception care is taking them through what pregnancy is all about before they even get into pregnancy.

Interviewer: What are the components of PCC?

P13: So we are talking about the pre, things needed to do, the woman needs to take folic acid to boost the immunity, you make sure that your HB is even enough active component because when you have low HB and you are pregnant you are at risk to loose the pregnancy, is a high risk therefore your HB, compatibility between you and your partner, I don’t know whether when you were going for your marriage you did the compatibility test but as a client if you come to me those are the things that I will make sure you go through them. Then you go through other tests like HIV, syphilis, sickle cell and those kind of things so that we know that both of you are even compatible to start a family, to start a baby because by the end of the day the fetus that is coming in the baby that is coming to the nation is not only for you the two of you is for the entire nation so we’ve to make sure everything is ok. Taking them through all these things, knowing their compatibility, their blood level; someone with O negative getting married to any blood group that is positive their first conception will stay but the subsequent ones might cause abortions. So, if your partner comes before and then you know all these things, there are things that they will put in place.

Interviewer: Which category of individuals require PCC?

P13: Everyone within their reproductive age, it is required to know about preconception care.

Interviewer: Probe: Prioritizing people

P13: Yes, those with special need, let’s say a client with HIV positive they need special care because they need to continue taking their drugs, change of medications making sure that their viral load is down so that it won’t affect the fetus coming. Then sickle cell clients because sickle patient going through pregnancy can also sometimes even you may lose your life through the pains and other things so when someone come for preconception care and this person is sickle patient you the person needs extra extra because of already condition that they have. Someone with already hypertensive client, diabetes client during pregnancy those conditions also goes high.

Interviewer: What are the benefits or outcomes of PCC?

P13: Ok, just as I was saying, those with special conditions maybe those don’t know the effects of whatever will happen during pregnancy but with the help of preconception care they will get the knowledge to understand their body well, the things that will happen and the reason for their treatment. Some people when you give them drugs is not because they don’t want to take but they don’t know the necessity of the drugs, so when they understand the reason for taking those drugs there is no way that they won’t take it. Am sickle patient my blood cells always breakdown before the end of the month and when am pregnant this baby this fetus needs more blood, so when this person becomes pregnant they need of increasing their drugs is more than that without the sickle condition so that you get more of the nutrients more than the others, her eating habit and everything, if you don’t take them through one-on-one personally they wouldn’t understand, is like you are treating them differently but when you let them understand their condition their situation they really appreciate it and it makes the work easier for both the client and relatives, yeah.

Interviewer: What are the standardized procedures on PCC from WHO, MOH, GHS, and CHAG that you know of?

P13: We’re doing focus antenatal care as in Ghana here and if you talk of focus antenatal care, we’re talking about the client individually per your condition per your reason we treat accordingly, so that’s the standards…. I have preconception care client that am taking them currently and they are too anxious to get pregnant. You see like the body is, we’re married so we need to get pregnant that’s not how pregnancy is supposed to be occurring. When you are married you need to enjoy your married life so that everything comes naturally, don’t be so conscious of your life, so you see where their mind is, that fact that the man is putting sperms inside the woman she is supposed to also get it no, it needs to move freely, when you are anxious your eggs will not fertilize well, yes, so you see this client of mine it will take me a longer period for her to get over with her anxiety, so that’s how it works, yes.

Interviewer: How do you receive clients seeking for PCC services in your facility? Probe:

P13: Am happy they always come to me! You see is happy you are able to help someone to get pregnant, is like you are part of the pregnancy during the journey till delivery you understand? Am helping you to get pregnant that means am part of the family, during pregnancy am taking care of this baby then God been so good after nine months am able to welcome this baby into this world, won’t you be happy? Yes, that’s how I feel, that’s how I welcome them when they come to me.

Interviewer: How do you provide PCC services to your clients?

P13: It depends on what they want to know, they client I was taking about came in with err he wants to know about my menstrual cycle, the husband came in to know her menstrual cycle how it works and I was like you can’t use my menstrual cycle to determine your wife own because we have different menstrual cycle depending on the individual, so I let him download the menstrual app on the phone and invited both the wife and the husband, then we started with the day of his wife menstrual cycle, her first date of menstruation, so have to explain the ovulation period, the free period, the emergency period, do you understand it?...on the calendar for them, the number of days that her free period may last, so let’s say that I have 3 free periods within my menstrual cycle, first week of menses then after 2 weeks I’ve extra five days free period, I was supposed to let them know that the first three days if she things she is having free period and have menses within the first three days she can get pregnant because the sperm has a life span of five days within her womb, so the free period is not just three there is an emergency days ahead which can cause her get pregnant, so in other for her to get the free period I have to take her back to her other five periods there so that if she wants to enjoy free sex she could have used that one instead of the first one, and it wasn’t only the wife it was both the husband and wife where they were able to calculate their free period, her menstrual length and everything.

Probe: other services….

P13: since they were eager to have babies and both of them were anxious, we need to do management on the anxiety, improve her folic acid intake, her vaccine and others. So, we manage according to the severity of the condition.

Interviewer: Where do you receive and provide PCC services to your clients?

P13: I said focus antenatal care so when you are taking of focus is just you and you client, one-on-one.

**DEMOGRAPHIC INFORMATION**

Interviewer: Can you share with me a brief information about yourself?

P14: Age: 29 years, gender: female, marital status: single, religion: Christian, education: diploma in midwifery, years of practice: 4 years, current grade: Senior Staff Midwife

**KNOWLEDGE, ATTITUDES, AND PRACTICES OF MIDWIVES ON PCC**

Interviewer: What is your understanding of PCC?

P14: Preconception care what I understand is that before you become pregnant what you go through before you become pregnant that is the preconception care, sometimes you do some antenatal class preconception class for them and other things.

Interviewer: What are the components of PCC?

P14: Ok, so some of the things we do for them are like we have to psyche them that pregnancy is not a disease is like a change a hormonal change in the system and is not permanent is temporal and some of them we advise them to take folic acid in other to prevent teratogenic effects and other things in pregnancy…

Probe about labs…

P14: Yes, yes we do, we do errm genotypes and errm blood groups you sometimes you’ve to know the genotype of the mother and the father the parents so that there won’t be any anti D issues like you the husband is a sickler, the husband is negative and the wife is positive and other things so we do all those things to let them know if there would be any effect before they conceive.

Interviewer: Which category of individuals require PCC?

P14: Ok, the category of people, new couples and those who have like nullips and are you there….so what I was saying, I said you do for those who have been in relationship and like they’ve not had any child you do for them and new couples too we do for them…….

Interviewer: Probe: Prioritizing people

P14: ok, ok I forgot and those who have also given birth to like abnormal children. What we do is that like people we give this conception like the care to or what…. ok, so people who have whose rhesus factor like the husband and the mother the wife the rhesus factor are incompatible we do for them too and if they have had previous like miscarriages and other things we also do for them.

Interviewer: What are the standardized procedures on PCC from WHO, MOH, GHS, and CHAG that you know of?

P14: Actually, I don’t think, I don’t think we have because I have never seen a guideline for preconception care, I have never seen it in my field of work. Since I not seen any preconception guideline what I do is that what I’ve learnt and the little knowledge I have been in a village because I started working, they’ve never provided any preconception guidelines to us.

Interviewer: How do you receive clients seeking for PCC services in your facility? Probe:

P14: Ok, I receive them warmly and reassure them that I’ll try my best to also help, because I cannot assure and like give you a straight like you will give birth too no, and if something goes wrong it would be like I didn’t do my work, so we assure them that we would try our best and with God things would be possible, and sometimes too we counsel them if we get to know that there is rhesus incompatibility we let them know we Let them know and if there is anti D that they should take before first conception they’ll take so that it will prevent subsequent miscarriages. You shouting would just aggravate everything, so we talk to them nicely so that they would also have some trust that things would work well. Because if someone comes to you and you shout on the person it looks like you’re chasing the person away, so when they come, we try and make things easy for them.

Interviewer: Where do you receive and provide PCC services to your clients?

P14: Aaa, is client centered so we don’t do it in the general way so is individual. Ideally you are supposed to have a room so that we do, but in our setting, we don’t have that errm facilities. So, what we do is that if the person comes to you, you schedule the person to your free time and their also free time so that they’ll come and you’ll have time for them, it won’t be in a rush so that sometimes you’re the only midwife at the facility so when you come and there are pregnant women there I won’t get time for you so what we do is that we tell them we ask of their free time and we also compare to our also free time then we invite them to come so that we will have enough time for them because there would be questions and answers.

Interviewer: Have you ever provided PCC to your clients? Explain your response

P14: The last preconception care I did for a couple it was for them they have given birth before but they were not conceiving anymore so they came to me and it was I helped them with their cycle, I helped the woman know her ovulation date and they were successful and she was taking I advised her to take folic acid and other things and if they had been smoking and other things they should avoid alcohol, so they started taking folic acid and I taught her cycle how to follow the cycle and she got to know here ovulation period day and she became successful that was the last I did. So, for her case, she has given birth before is just that she was not conceiving anymore so they were worried and I talked to them and they were able to they went through successful. She has delivered and I’ve left Kitari I don’t know what is going on there again actually I didn’t take their contact because that time in Kitari we were not having network.
