# Supplementary material for: An exploratory study on midwives’ perceptions of the availability of preconception care in health facilities in the Kpandai District of Northern Ghana
Source: PLOS Glob Public Health. 2025 Aug 18;5(8):e0003850. doi: 10.1371/journal.pgph.0003850 (PMC12360517; doi:10.1371/journal.pgph.0003850)
Supplement: Attachment — Submitted filename: AUTHORS RESPONSE.docx [file pgph.0003850.s003.docx]

**AUTHORS RESPONSE**

Manuscript number: PGPH-D-24-02270

| **Editorial Comments** | **Authors Action** |
| --- | --- |
| Justification for Sampling Methods: Please provide a clear and detailed justification for the use of both purposive and random sampling techniques within your qualitative study. Explain how these methods align with your study's objectives and contribute to the depth and breadth of the data collected. Additionally, discuss the rationale for integrating these sampling approaches and how they complement each other in achieving a comprehensive understanding of the research phenomena. | Comments addressed on page 7, lines 150 – 180. Justification and rationale for the use of both random and purposive sampling for this qualitative study have been provided. |
| Description of Manual Thematic Coding: The description of your manual thematic coding process requires further elaboration. Provide a step-by-step explanation of how you approached coding the data manually. For instance: Detail how you identified themes or patterns, specify any frameworks, criteria, or guidelines used during the coding process and describe how codes were reviewed, refined, and organized into broader themes.  To strengthen this section, consider referencing articles or methodologies that offer established and detailed processes for manual coding in qualitative research. For example: https://doi.org/10.1177/16094069241299. This will enhance the clarity and credibility of your methodology. | A step-by-step explanation of how the manual thematic coding and analysis is provided on page 10, lines 212 – 230. These were supported by relevant articles; page 32, lines 651 - 662. |
|  |  |
| **Reviewer comments** |  |
| The authors can be consistent in using terms such as "good attitude" or "positive" vs. favorable. | The authors considered the consistent use of “good attitude” throughout the manuscript. |
