# Supplementary material for: An exploratory study on midwives’ perceptions of the availability of preconception care in health facilities in the Kpandai District of Northern Ghana
Source: PLOS Glob Public Health. 2025 Aug 18;5(8):e0003850. doi: 10.1371/journal.pgph.0003850 (PMC12360517; doi:10.1371/journal.pgph.0003850)
Supplement: Attachment — Submitted filename: AUTHORS_RESPONSE_auresp_2.docx [file pgph.0003850.s004.docx]

**AUTHORS RESPONSE**

Manuscript number: PGPH-D-24-02270 R1

Thank you very much to the Chief Editor and reviewers for your interest in this manuscript. Please find below the reviewers' comments and the actions undertaken by the authors.

| **Editorial Comments** | **Authors Action** |
| --- | --- |
| 1. Please review your reference list to ensure that it is complete and correct. If you have cited papers that have been retracted, please include the rationale for doing so in  the manuscript text, or remove these references and replace them with relevant current references. Any changes to the reference list should be mentioned in the rebuttal letter that accompanies your revised manuscript. If you need to cite a retracted article, indicate the article’s retracted status in the References list and also include a citation and full reference for the retraction notice. | Thank you for your feedback on the completeness and correctness of the reference list. We have rechecked the reference list and ensured its correctness and completeness. The reference for “Fekadu GA, Chojenta C, Barreix M, Loxton D. Role of women’s autonomy and their negotiation power on maternal healthcare utilization in Ethiopia. BMC Pregnancy Childbirth. 2021;21(1):1–13” has been removed from the reference list. |
| 2. Please simplify the language suitable for a science journal, not a newspaper style. | Thank you very much for your feedback. The language throughout the entire manuscript has been revised to enhance its suitability for publication in a scientific journal. |
| 3. "Midwives perceptions of the availability of preconception care" appears to be distal indicator. Ideally if it has any value for programs, it should start with an inclusive review of all aspects of preconception care availability not just midwives "perception". In addition to the midwives, there are nurses, and other clinical service providers, ministry of health guidance, fidelity of the integration of such guidance, and health care seeking behaviors of women of childbearing age. If health care seeking prior to conception is not a practice, how would midwives intervene? Do the midwives go and talk to each women of childbearing age? Or do you have a preconception counseling at the time of marriage registration as it was in China, leading to the studies on folic acid supplementation? So, what good is to study the perceptions of a fraction of service providers, when we do not know if all other elements of preconception care are in place? | Thank you very much for your constructive feedback. We would have liked to expand this research to cover the entire country and include all relevant stakeholders, such as government officials, representatives from the Ministry of Health, Ghana Health Service, the Nursing and Midwifery Council of Ghana, principals of nursing and midwifery training institutions, physicians, and other healthcare providers. This broader scope would have allowed for a more comprehensive report to inform policies and programs in both academia and clinical practice, ensuring that activities preceding conception are properly monitored and managed to prevent birth defects, labor complications, and maternal and newborn mortality.  However, we did not receive funding to scale up the study to include all these stakeholders. Nevertheless, it was our intention to start at the grassroots level, focusing on the area where services are currently provided. In the future, we hope to apply for domestic or international research grants to expand the study, incorporating data particularly from service providers such as midwives, who are directly responsible for the care of women before and after conception. |
